# Impact of memory T cells on SARS-CoV-2 vaccine response in hematopoietic stem cell transplant

**DOI:** 10.1371/journal.pone.0320744

**Published:** 2025-04-28

**Authors:** Jennifer VanOudenhove, Yuxin Liu, Raman Nelakanti, Dongjoo Kim, Emma Busarello, Natalia Tijaro Ovalle, Zhihong Qi, Padmavathi Mamillapalli, Alexa Siddon, Zhiliang Bai, Alfredo Axtmayer, Cheryl Corso, Shalin Kothari, Francine Foss, Iris Isufi, Toma Tebaldi, Lohith Gowda, Rong Fan, Stuart Seropian, Stephanie Halene

**Affiliations:** 1 Section of Hematology, Department of Internal Medicine and Yale Cancer Center, Yale University School of Medicine, New Haven, Connecticut, United States of America; 2 Department of Biomedical Engineering, Yale University, New Haven, Connecticut, United States of America; 3 Department of Cellular, Computational and Integrative Biology (CIBIO), University of Trento, Trento, Italy; 4 Department of Internal Medicine, Yale University School of Medicine, New Haven, Connecticut, United States of America; 5 Department of Laboratory Medicine, Yale University School of Medicine, New Haven, Connecticut, United States of America; 6 Bone Marrow Transplant and Cellular Therapy Program, Yale New Haven Hospital and Yale Cancer Center, New Haven, Connecticut, United States of America; 7 Department of Pathology, Yale University School of Medicine, New Haven, Connecticut, United States of America; Augusta University, TAIWAN

## Abstract

During the COVID-19 pandemic, hematopoietic stem cell transplant (HSCT) recipients had elevated mortality rates from SARS-CoV-2 infection, ranging between 10–40%. SARS-CoV-2 mRNA vaccines are important tools in preventing severe disease, yet their efficacy post-transplant remains unclear, especially in patients subjected to myeloablative chemotherapy and immunosuppression. We evaluated humoral and adaptive immune responses to the SARS-CoV-2 mRNA vaccination series in 42 HSCT recipients and 5 healthy controls. Post-vaccination responses were assessed by anti-spike IgG and nucleocapsid levels, and antigen specific T cell activity. Immune profiling was performed using clinical flow and mass cytometry. Patients were selected based on humoral and cellular responses for single-cell RNA with TCR and BCR sequencing. Our studies revealed defects in memory T cells that correlated with an absence of cellular response despite nearly universal humoral response. Several patients with a robust antibody response developed COVID-19 infection, but none developed severe disease or died from the infection.

## Introduction

Hematopoietic stem cell transplant (HSCT) recipients are at an increased risk for poor outcomes from severe acute respiratory syndrome coronavirus 2 (SARS-CoV-2) infection due to an impaired immune response. A combination of factors contribute to a compromised immune system in HSCT recipients, including history of prior hematologic malignancy, treatment with myeloablative or lymphodepleting chemotherapy, and usage of immunosuppression or further maintenance therapy following transplant [1]. Data suggest that all-cause mortality rates from COVID-19 infections in HSCT recipients range between 10–40% [2–6].

Given the increased risk for severe COVID-19 infection and mortality among HSCT patients, proactive infection mitigation is paramount. SARS-CoV-2 mRNA vaccines, including Pfizer’s BNT162b2 and Moderna’s mRNA-1273, were made available under Emergency Use Authorization (EUA) in December 2020 after they showed a 95% and 94.1% efficacy, respectively, in preventing severe symptomatic COVID-19 infection [7,8]. However, these initial trials excluded immunocompromised patients from their enrollment, leading to insufficient data on the efficacy of vaccination in those with immune deficiencies. Despite this uncertainty, vaccination in immunocompromised populations, including HSCT recipients, commenced in 2021. Initial studies were optimistic, showing that sero-conversion in response to SARS-CoV-2 vaccination following allogeneic stem cell transplantation was between 76–83% depending on the study [9,10]. In autologous stem cell transplant recipients, Salvini et al. showed that 87% of patients developed a humoral immune response [11]. These results were further corroborated by other large cohort studies in the subsequent years [12–15]. However, in addition to humoral immunity, cellular immune determinants contribute to SARS-CoV-2 infection clearance [16]. Given the changes in the cellular immune system following HSCT and exposure to immunosuppressive agents, less is known regarding the cellular immune response following SARS-CoV-2 mRNA vaccination [17].

Here we report the results from a 22 month long prospective observational study of the immunologic efficacy and comprehensive immune profiling of the SARS-CoV-2 vaccination in 42 hematopoietic stem cell recipients and 5 healthy controls. Single cell RNA with TCR and BCR sequencing was performed on patient PBMCs for a more in-depth exploration of possible mechanisms behind response or lack of response to SARS-CoV-2 vaccination following HSCT.

## Results

### Cohort clinical characteristics

Our study included 42 HSCT patients (37 allogeneic and 5 autologous), and 5 healthy controls as outlined in Table 1. The median age of the allogeneic HSCT group was 65 years old, with ages ranging from 25 to 78. Males represented 69.4% of this group. The majority of the individuals in the cohort were allogeneic transplant recipients (alloSCT) diagnosed with a myeloid malignancy (81%). The median time from transplant to vaccine (TTV) in alloSCT recipients was 19 months, and 40.5% of the alloSCT patients began their SARS-CoV-2 vaccination less than 12 months after transplant. History of chronic graft versus host disease (cGVHD) was found in 56.8% of alloSCT patients, and 54.1% were on immunosuppressants (S1 Table). Among those alloSCT recipients vaccinated more than 12 months after their transplant, 68% had active cGVHD at the time of their vaccination, compared to 25% of alloSCT recipients who started their vaccination series less than 12 months post-transplant. Among the five autologous stem cell transplant recipients (autoSCT) in the cohort, most had been diagnosed with multiple myeloma with a median TTV of 4 months. No patient received therapeutic anti SARS-COV2 monoclonal antibodies during the time samples were collected to analyze antibody and T cell responses.

**Table 1:** Overall HSCT cohort characteristics.

	Allogenic (n = 37)	Autologous (n = 5)	Control (n=5)
**Age at Vaccine (median, range)**	65 (25-78)	60 (54-72)	54 (35-85)
**Biological Sex (Male)**	25 (69.4%)	1 (20.0%)	3 (60%)
**Hematologic Malignancy**			
AML/MDS/MF	30	0	0
ALL	4	0	0
Lymphoma	2	1	0
Multiple Myeloma	1	4	0
**Median time to vaccine from transplant (range, months)**	19 (7-79)	4 (4-10)	x
**Vaccination ≤12 mo from transplant**	15 (40.5%)	4 (80%)	x
**cGVHD at time of vaccine**	21 (56.8%)	0	0
**On immunosuppression**	20 (54.1%)	0	0
**Lymphopenia (ALC <1x10^4/ul)**	9 (24.3%)	4 (80%)	0
**Documented COVID prior to vaccine**	3 (8.1%)	0	0
**Developed COVID post vaccine**	14 (37.8%)	1 (20%)	1 (20%)

### Robust antibody response achieved with three doses of the SARS-CoV-2 vaccine

**Fig 1:**
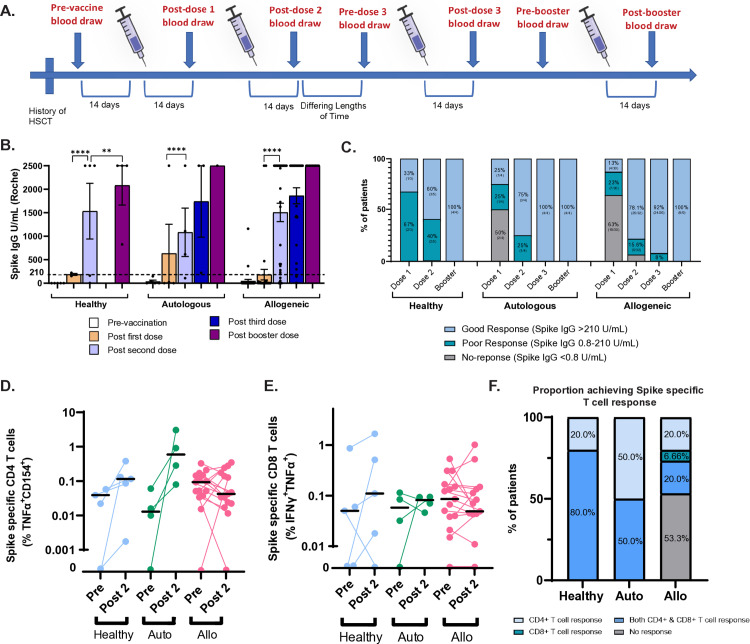
A. Study Design. B. SARS-CoV-2 Spike antibody titers were assessed using the Roche Elecsys anti-SARS-CoV-2 Spike immunoassay pre-vaccination and after each subsequent dose. C. Proportion of patients in each cohort who achieved a good, poor, and no antibody response. Mann-Whitney test continuous variables with Bonferroni correction for multiple comparisons were performed, with two-sided p-values ≤ 0.05 considered statistically significant with p ≤ 0.01 = ** and p ≤ 0.0001 = ****. D. Flow Cytometry analysis was performed on PBMCs taken pre-vaccination and following 2nd vaccine dose (5 controls, 4 autoSCTs and 16 alloSCTs). Percent of spike reactive CD4 cells pre and post dose 2 of the vaccine over vehicle stimulated cells by markers TNFα and CD154. E. Same as D, except for CD8 cells and markers IFNγ and TNFα. F. Proportion of patients in each cohort who achieved a spike-specific T cell response. Wilcoxon signed-rank tests were performed, with a p-values ≤ 0.05 considered statistically significant.

Peripheral blood mononuclear cells (PBMCs) and serum from HSCT patients and healthy controls were prospectively collected before and after each dose of the SARS-CoV-2 vaccine (Fig 1A). Serological testing for SARS-CoV-2 Spike and Nucleocapsid antibodies revealed no significant differences in antibody levels between groups at any vaccination stage (Fig 1B and 1C). However, after the first mRNA vaccine dose, 25% of autoSCT and 13% of alloSCT patients exhibited an effective spike antibody response (defined as anti-Spike IgG titer > 210 U/mL, based on the value given by the FDA in the EUA for high titer convalescent plasma levels via the same Roche assay), in contrast to 33% of healthy controls. Following a second dose, 75% of autoSCT, and 78.1% of alloSCT patients achieved a robust spike antibody response. In all cohorts, three doses of the vaccine resulted in a robust response in 92–100% of patients. Of note, our healthy cohort was only composed of five individuals spanning the age range of 35–85, including two individuals who were 80+ years old and, as has been previously reported, required a booster to achieve an effective response [18]. Among three patients with previous COVID-19 infections, two tested positive for nucleocapsid antibodies before vaccination, and the third’s infection was nearly a year past and potentially had lost seropositivity [19]; all other patients were negative for Nucleocapsid antibody consistent with low infection rates in patients who followed strict isolation practices.

### AlloSCT patients have a low rate of SARS-CoV-2 antigen specific T cell response

**Fig 2:**
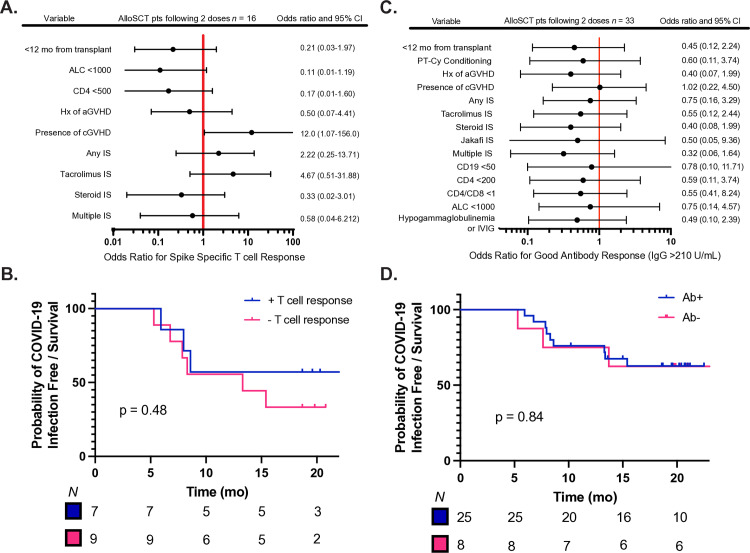
Forest Plots demonstrating clinical variables of interest and their odds ratios of developing a positive T cell response (panel A) or a good (>210 AU/mL) antibody titer (panel C) among allogeneic stem cell recipients. Gehan-Breslow-Wilcoxon tests were performed to test if having a SARS-CoV-2 specific T cell (panel B) or a significant antibody (panel D) response resulted in a difference in probability of COVID infection and mortality. IS stands for immunosuppression.

Since most patients had an adequate humoral response after the second vaccination dose, we chose to test SARS-CoV-2-specific T cell function via an antigen-specific T cell activation assay on PBMCs of 24 patients (16 alloSCT, 4 autoSCT, 5 control) before and after the second vaccine dose. The percentage of activated T cells was reported as a percentage of the sample displaying the activation markers above unstimulated cells (Fig 1D and 1E). A positive spike-specific T cell response was defined as having either an increase of double positive TNFα+CD154+ CD4 T cells between pre vaccine and post second dose or an increase of double positive IFNγ+TNFα+ CD8 T cells (Fig 1F). In total, 46.7% of alloSCT patients demonstrated a positive spike-specific T cell response compared to 100% of our healthy and autoSCT cohorts. When comparing the percentage of spike specific CD4 and CD8 T cells before and after 2 doses of vaccination in each cohort, we observed a slight, but non-significant (by Wilcoxon signed rank-test) increase in the average percentage of antigen-specific T cells in CD4 T cells in healthy and autoSCT recipients that wasn’t seen in alloSCT recipients. Statistics were hampered by the low magnitude of response in the presence of a small pre vaccination population of reactive T cells. By univariate analysis of clinical variables and their relationship with a positive T cell response, cGHVD demonstrated an odds ratio of 12.0 that was statistically significant (95% CI = 1.07–156) using Fisher exact tests (Fig 2A). Gehan-Breslow-Wilcoxon tests did not show a significant difference in the probability of developing COVID-19 infection and mortality among patients who did or did not develop a cellular immune response or an antibody response to the vaccine (Fig 2B and 2D) and neither uni- nor multivariate analysis of clinical variables identified statistically significant relationships with an antibody response (Fig 2C, S2 Table).

### Immune profiling pre-vaccination reveals deficits in T cell populations

**Fig 3:**
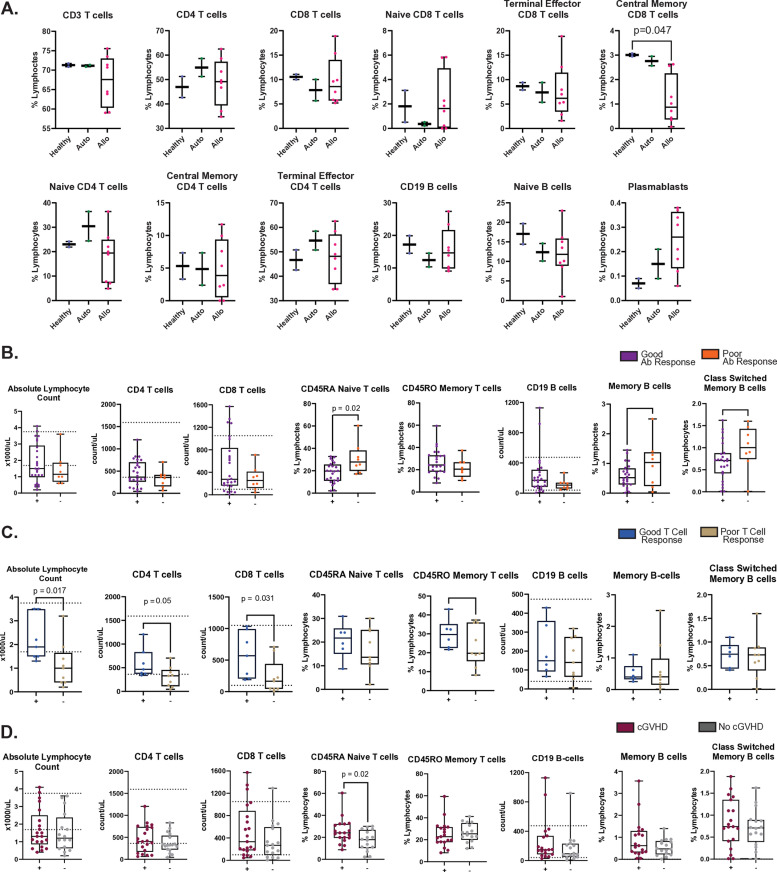
A. CYTOF results showing cell types presented as percentage of lymphocytes separated by transplant type. B. Clinical pre-vaccination immunodeficiency flow panel results separated based on antibody response of allogeneic patients. C. Clinical pre-vaccination immunodeficiency flow panel results separated based on SARS-CoV-2 specific T cell stimulation assay response in allogeneic patients. D. Clinical pre-vaccination immunodeficiency flow panel results separated based on cGVHD in allogeneic patients. Mann-Whitney test for continuous variables were performed, with two-sided p-values ≤ 0.05 considered statistically significant. Reference ranges for the variables that have them are indicated by the dotted lines.

Mass Cytometry was carried out on PBMCs prior to vaccination and after the first and second doses in 2 healthy individuals, 2 autoSCT patients, and 8 alloSCT patients (S3 Table). Among this group, 58.8% of patients developed a good antibody response and 72.3% developed a positive T cell response after two vaccine doses. Immune cell subsets at baseline were examined amongst the three different groups before vaccination (Fig 3A). The significant finding from this data was that there were fewer central memory CD8 T cells in alloSCT patients compared to healthy controls (healthy: 3.01%, alloSCT, 0.87%;p = 0.047) using a one-way ANOVA analysis with Bonferroni correction for multiple comparisons. No significant variations in immune cell subsets were observed between transplant cohorts and healthy controls after the first or second vaccination.

Additionally, a thorough evaluation of the cellular immune repertoire was conducted by flow cytometry for 39 out of 42 HSCT recipients prior to vaccination. We examined whether any immune cell subset present prior to vaccination was associated with antibody response in allogeneic transplant patients after two doses of the SARS-CoV-2 mRNA vaccine (Fig 3B and S1 Fig). Allogeneic transplant recipients with poor antibody response had a significantly higher percentage of CD45RA naïve T cells at baseline compared to those with a good antibody response (poor responders: 27.8%, good responders: 19.68%; p = 0.02). Additionally, an evaluation of the relationship between baseline immune subsets and antigen specific T cell response in allogeneic transplant patients was conducted (Fig 3C and S1 Fig). Patients who did not develop a SARS-CoV-2 spike-antigen specific T cell response had a lower absolute lymphocyte count (ALC) (responders: 1.9*10^3 cells/µL, non-responders: 1.0*10^3 cells/µL; p = 0.017) with decreased counts of CD4 T cells (responders: 465 cells/µL, non-responders: 329 cells/µL; p = 0.05) and CD8 T cells (responders: 569 cells/µL, non-responders: 166 cells/µL; p = 0.031). Since the presence of cGVHD (Fig 2A) was shown to be positively correlated with a good T cell response, we examined whether cGVHD was associated with any immune cell subset prior to vaccination in allogeneic transplant patients after two doses of the SARS-CoV-2 mRNA vaccine (Fig 3D and S1 Fig). AlloSCT patients had a significantly higher percentage of CD45RA T cells (cGVHD: 24.0%, no cGVHD: 17.86%; p = 0.02).

### Single cell RNA-sequencing reveals defects in CD8 central and effector memory T cells associated with poor T cell response to SARS-CoV-2 vaccination

**Fig 4:**
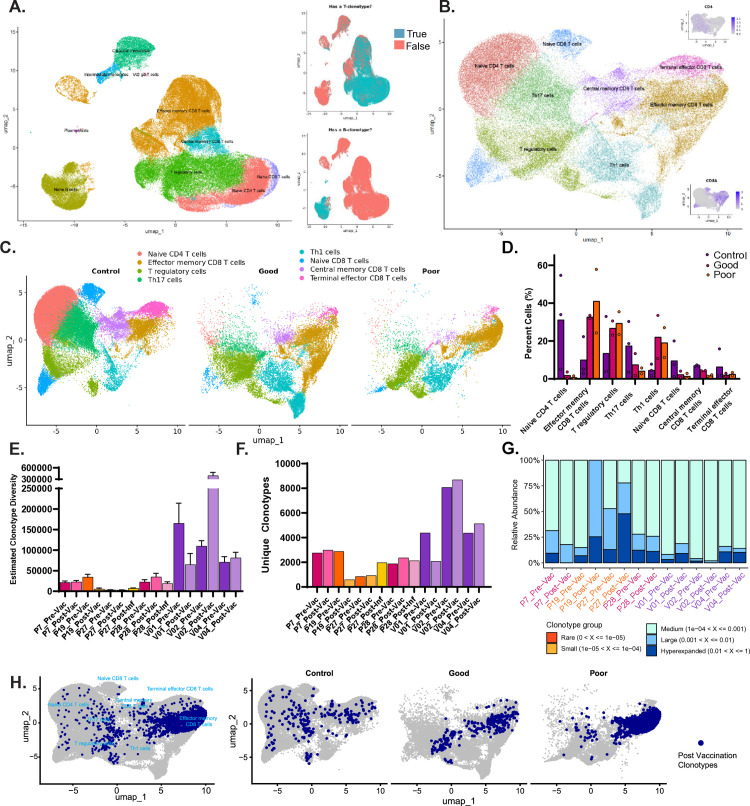
Single cell RNA sequencing of patient samples and TCR clonotype analysis. A. Seurat clustering annotated by reference annotation “MonacoImmuneData”, subpanels on the right (top) cells in teal are positive for having a T cell clonotype, and cells colored in red do not have a T cell clonotype attributed from the T cell sequencing, (bottom) same as top, except for B cell clonotypes. Cells with high mitochondrial reads or low or extremely high RNA counts were filtered out. B. Data was filtered to include only T cell clusters and excluded two post-COVID-19 infection samples. UMAP projection of the T cells re-clustered and re-annotated as before with markers *CD4*, and *CD8*. C. Cells from the UMAP projection in D were split by T cell response and colored by cluster annotation. D. Quantitation of C, where the percentage of each individual’s cells in each cluster was calculated (Healthy n = 3, Good n = 2, Poor n = 2) and plotted on a bar graph. E. TCR repertoire diversity using the Chao1 estimator, the calculated value is plotted as a bar graph displaying the calculated 95% confidence interval. Healthy donors are colored in purple, good responders are in pink and poor responders are in orange and in D. F. A bar graph displaying the number of unique clonotypes in each sample sequenced, colored as in D and E. G. Summary proportion of clonotypes with specific frequencies by sample. The sample labels follow the color scheme from E. H. Top clonotypes found to be unique to post vaccination when compared to pre vaccination for each individual were mapped back to the cells in the T cell UMAP both in aggregate (left) and the UMAP with cells divided by T cell response in the antigen specific T cell assay cohort (right).

To better understand predictors of cellular responses to SARS-CoV-2 mRNA vaccines in SCT recipients, we performed 10X single cell RNA-seq (scRNA-seq) combined with T Cell Receptor (TCR) and B Cell Receptor (BCR) sequencing on pre- and post-vaccination (2^nd^ dose) samples, as well as post-infection samples when available, from 4 alloSCT recipients and 3 healthy controls. These subjects were chosen to include both good and poor humoral and T cell responders to vaccination. The clinical characteristics of the patients selected for single cell RNA seq are provided in S2 Fig 2A. From the seven individuals sequenced, 117,492 cells passed quality control to exclude doublets, cells with high mitochondrial reads, and feature counts below or above what would be expected for a single cell (S2 Fig 2B-2C). High quality cells were clustered based on their gene expression, and each cluster was assigned a cell type identity based on reference annotation dataset (“MonacoImmuneData”) (Fig 4A) [20].The presence or absence of a T or B clonotype in the TCR and BCR cell sequencing was used to further support the cluster identification.

The T cells were then analyzed separately, re-clustered, and re-annotated using fine labeling from the same reference annotation (Fig 4B). The expression of T cell markers *CD4* and *CD8*, is shown to support cluster annotation along with a heat map of the top marker genes (Fig 4B and S2 Fig 2D). To determine whether changes in T cell frequencies were evident in good versus poor responders compared to healthy controls (Fig 4C), we calculated the percentage of cells in each cluster for each individual sequenced (Fig 4D and S2 Fig 2E). All alloSCT recipients displayed decreased naïve CD4+ T cells, naïve CD8+ T cells, Th17 cells, and terminal effector CD8 T cells compared to healthy controls. AlloSCT recipients with a good T cell response, like healthy controls, had more memory CD8+ T cells compared to alloSCT recipients with a poor T cell response. Intriguingly, these scRNA-seq data are supported by the CYTOF analysis performed in a larger number of our patients. AlloSCT recipients who demonstrated either a good or poor T cell response had more Th1 and T regulatory cells than healthy individuals. Poor responders had the highest level of effector memory CD8+ T cells when compared to good responders and healthy controls. Gene set enrichment analysis comparing T cell gene expression post vaccination in good versus poor T cell responders revealed enrichment for a T memory cell signature in good responders (S1 Fig 1F and S4 Table) emphasizing the differentially expressed T memory marker genes *CCR7*, *LEF1*, *IL7R*, and *TCF7*.

Each sample’s T cell repertoire and diversity were further examined by using the corresponding data from the TCR sequencing. The Chao1 estimator function, a nonparametric asymptotic estimator of species richness, was utilized to calculate the clonal diversity on the amino acid level for each individual at each vaccination timepoint (Fig 4E). Then we determined the total number of unique clonotypes for each individual at each time point (Fig 4F). Next, we looked at the proportion of repertoire for each sample occupied by clonal groups with specific abundances (Fig 4G). First, as expected, healthy donors had more clonal diversity in comparison to HSCT patients. Specifically, individual P27, followed by P19, had the lowest post vaccination clonotype diversity, unique clonotypes, and percentage of clontype groups that were large or hyperexpanded. Interestingly, individual P27 had both poor antibody response and poor antigen specific T cell response, while P19 was able to mount an antibody response but not an antigen specific T cell response. Individuals P7 and P28, who had both good antibody and T cell responses, had higher clonotype diversity and number of unique clonotypes than the poorer responders, but not as high as the control healthy individuals.

Finally, the top clonotypes for each individual were queried for uniqueness between the pre and post vaccination time points, as clonotypes that appear post vaccination could be related to the vaccination event, and 256 unique clonotypes were identified (S5 Table). This list was then annotated using a database, TCRMatch, to determine the clonotypes’ potential predicted targets [21]. Of the 109 out of 256 clonotypes that matched with an entry in the annotation database, 67 were predicted to target a portion of SARS. The unique post vaccination clonotypes were then mapped back to the cells in the T cell UMAP (Fig 4G). When overlayed onto the T cell UMAP there was a noticeable accumulation of cells in the effector memory CD8+ T cells cluster. To determine whether there was any bias in which patients these cells were from, we split the overlay according to T cell response. In control individuals the post vaccination clonotypes were evenly distributed across the different cell types. However, in HSCT patients, the distribution of post vaccination clonotypes was much more restricted. Between the good and poor T cell responders, there was an even more predominate concentration of post vaccination clonotypes in the effector memory CD8+ T cell cluster of the poor responders. This suggests a defect in the T cells functional differentiation dynamics, potentially preventing cells from reaching the CD8+ central memory or terminal effector state.

We performed parallel investigations on B cell populations in the scRNA sequencing data. We could not identify differential trends in B cell populations between patient cohorts or differences in anti-Spike protein antibody response reflected in the uniformly appropriate humoral response in healthy controls and our alloSCT recipients (S2 Fig 2G-2I).

### Clinical outcomes

As of the last data update on November 26, 2022, 14 out of 37 alloSCT recipients (37.8%) and 1 out of 5 autoSCT patients (20%) had developed a COVID-19 infection. Among the 15 affected patients, two were admitted to the general medicine floor in the hospital, with no instances of ICU admissions or COVID-19 related fatalities. Three individuals passed away post-vaccination, but their deaths were not related to COVID-19 infection or vaccination complications. A Kaplan Meier analysis revealed no significant disparity in COVID-19 infection rates between patients with and without a strong antibody response, as visualized in Fig 2C. Importantly, no adverse reactions to the vaccine were reported.

## Discussion

Our findings provide a detailed analysis of the humoral and cellular immune response in allogeneic stem cell transplant recipients to the initial SARS-CoV-2 mRNA vaccines and offer insights that may influence clinical decision making for vaccination in this population. Our studies highlight a diminished antibody response in transplant recipients relative to healthy controls during the initial vaccine doses, most markedly after the first dose. Nevertheless, the introduction of a third standard dose, accompanied by subsequent boosters, markedly uplifts the proportion of good responders, aligning it with the response seen in healthy individuals.

Vaccination efficacy is often compromised in allogeneic recipients early after transplantation and routine revaccination is typically postponed at least 10–12 months post transplantation. We have shown that selected patients can respond at an early timepoint post-transplant to SARS-CoV-2 vaccination. This contrasts with other studies that have demonstrated that early vaccination from time of transplant (<12 months) and immunosuppression have been associated with poor serologic responses to vaccination [22,23]. Instead, in our cohort patients vaccinated on immunosuppression or that were <12 months following transplant achieved similar antibody responses to their counterparts. However, it should be noted that in our cohort, patients selected for vaccination less than 12 months from transplant were purposefully those patients off or almost off immunosuppression and without GVHD, which could be contributing to the lack of association between time from transplant and antibody response.

Beyond the anti-spike antibody response, the SARS-CoV-2 mRNA vaccine has also proven effective in eliciting potent T cell reactions in healthy subjects. Vaccine-induced CD8+ T effector cells may be detected as early as 10-days after the primary dose [24]. Our immune profiling indicates that HSCT patients who did not exhibit a spike-specific T cell response had reduced lymphocyte counts, including both CD4 and CD8 T cells, and fewer CD45RO+ memory T cells compared to those who did exhibit a spike-specific T cell response. This is further corroborated by our scRNA-seq data that demonstrates a larger CD8 central memory T cell compartment and higher memory T cell gene expression in the healthy control and good T cell responding alloSCT patients compared to the non-responders. The central memory T cell clusters in the post-vaccine samples appear stable from their pre-vaccine timepoint, which is consistent with prior data showing that a booster dose conserves the memory T cell pool [25]. Additionally, we found an accumulation of post vaccination specific clonotypes in the effector memory CD8+ T cell cluster is most pronounced in the poor responder cohort, which when combined with the T cell activation assay, suggests an additional functional defect in the effector memory T cell compartment. While single cell transcriptomic analysis did not reveal any statistically significant expression differences within the effector memory T cell cluster between cells from patients in the good vs poor cohort this could point to a post transcriptional mechanism that could warrant further study.

While we cannot say for certain that the donors of the transplant patients used in the single cell analysis were completely naïve to SARS-CoV-2, due to the times of transplant product harvesting (before early April of 2020) we can be assured that they are naïve to the vaccination. Due to the lack of pre vaccination antigen specific T cell response in these patients we would be led to believe that we are not observing transplanted response. We cannot determine, however, if the T cells that are generating a response come from naïve T cells adoptively transferred from the donor, or from CD34+ derived newly generated T cells in the transplant recipient, which would be of interest in the larger context of post-HSCT immune development. A T cell response was positively associated with the presence of cGVHD. All the patients in the scRNA-seq cohort that were good T-cell responders also had cGVHD. In future analyses, with larger cohorts, it could be of interest to specifically assess T cell responses to vaccination in patients with and without cGVHD.

Our study offers a comprehensive evaluation of the immune status of transplant recipients before and after vaccination forging a path for subsequent scrutiny into immune reconstitution and vaccine responses in this demographic. Although specialized analytical techniques, such as mass cytometry and single-cell sequencing, are not available in routine clinical care, we have demonstrated that there is concordance between clinical flow cytometry, mass cytometry, and single-cell sequencing. Therefore, more accessible clinical evaluations, such as absolute lymphocyte count and routine lymphocyte subset flow cytometry delineating CD4+ and CD8+ T cells, could serve as proxy indicators for a stem cell transplant recipient’s likelihood to develop a humoral and cellular immune response to the SARS-CoV-2 vaccine.

### Limitations of the study

We recognize the limitations inherent in our study, particularly its modest sample size and diverse patient group in terms of diagnosis, treatments, and age. Race, ethnicity, and socioeconomic status were not well documented for all patients in the medical record and therefore were not included in this analysis. It’s crucial to understand that the majority of COVID-19 cases in our cohort arose during the Omicron variant surge, where even vaccinated healthy individuals faced high breakthrough infection rates due to the original vaccination series targeting the original Wuhan variant.

## Materials and methods

### Resource availability

#### Lead contact.

Further information and requests for resources and reagents should be directed to and will be fulfilled by the lead contact, Stephanie Halene (Stephanie.halene@yale.edu).

#### Materials availability.

This study did not generate new unique reagents.

#### Study participant details and ethical approval statement.

This cohort includes patients receiving a transplant of adult stem cells and receiving mRNA vaccine(s). Patient clinical data including age, sex, diagnosis, type and timing of transplant, immunosuppression use, history of chronic graft versus host disease (cGVHD), and COVID-19 infection before or after vaccination were collected from the electronic medical record and are reported in summary in Table 1, this data was accessed for this paper between 15/02/2021 and 30/11/2022. Race, ethnicity, and socioeconomic status were not well documented for all patients in the medical record and were not included in this analysis. Samples that had been banked in the Yale Specimen Repository for Hematologic Diseases between 10/11/2020 and 28/07/22 were used in this study. The majority of samples and data used in this paper covered the COVID waves from original Wuhan strain through Omicron BA.5. A summary of the samples tested in each assay per patient is included in S6 Table. This study was conducted according to the Declaration of Helsinki and was approved by the Institutional Review Board at Yale University and conducted under HIC#1401013259 (Specimen Repository for Hematologic Diseases) to which all participants gave written informed consent.

### Method details

#### Sample collection.

Peripheral blood was collected in red top tubes containing no anti-coagulant, allowed to clot, then centrifuged at 1500g for 10 minutes at 4°C, the supernatant was then collected as serum. Peripheral blood samples collected in lavender EDTA coated tubes were layered over Ficoll and separated by density gradient centrifugation at 1800RPM for 20 minutes with the brake off. Peripheral blood mononuclear cells (PBMNCs) were collected from the buffy coat layer, washed with PBS, counted, and then cryopreserved in FBS with 10% DMSO.

#### Serologic antibody measurement.

Spike and Nucleocapsid antibody titers were assessed using the Roche Elecsys anti-SARS-CoV-2 Spike immunoassay (Spike), Roche Elecsys® SARS-CoV-2 Antigen assay (Nucleocapsid) and a novel microfluidic serologic device [26]. A robust spike IgG antibody response was defined as >210 AU/mL for the Roche assay and >38 BAU/mL in our novel device. The Roche Nucleocapsid assay is read out with an Index value that is then categorized as Reactive for an Index value >1.

#### T cell activation assay.

Antigen-specific T cell stimulation was performed on PBMCs taken pre-vaccination and following second vaccine dose with the Miltenyi Biotec SARs-CoV-2 Prot_S T Cell Analysis Kit and Peptivator® Prot-S Peptide Pool. The peptide pool contains the immunodominant sequences to the Wuhan SARS-CoV-2 spike protein (aa 304–338, 421–475, 492–519, 683–707, 741–770, 785–802, and 885–1273). Cells were thawed from liquid nitrogen storage and rested overnight at 37°C. PBMCs were plated with 1*10^6 cells per well and then stimulated according to the manufacturers protocol for 2 hours before addition of Brefeldin A to inhibit exocytosis of activation signaling proteins, and then stimulation continued for an additional 4 hours. Cells were stained for intra and extracellular activation markers (TNFα, IFNγ, CD154/CD40L), as well as T cell markers (CD3, CD4, CD8). Flow cytometry analysis was performed using a BD Symphony Cell Analyzer and analyzed using Flow Jo (v10). Cells were gated based on forward and side scatter to gate out debris, then doublet discrimination was performed. CD3+ cells were gated for and then separated based on CD4 and CD8 expression and then the levels of TNFα and CD154 in CD4+ T cells was assessed as were the levels of IFNγ and TNFα in CD8 T cells. Wilcoxon signed-rank test was performed to assess statistical significance.

#### Immune Profiling.

A clinical flow multiparametric flow cytometry designed to evaluate immunodeficiency syndromes was performed by YNHH Department of Laboratory Medicine on whole blood samples of our transplant cohort prior to first vaccine dose. Flow cytometric immunophenotyping used a panel of fluorescently labeled murine IgG monoclonal antibodies (see below).

**Table:** 

Antigen	Fluorochrome	Clone	Vendor
CD45	PerCP-Cy5.5	2D1	BD Biosciences
CD3	FITC	SK7	BD Biosciences
CD4	PE-Cy7	SK3	BD Biosciences
CD8	APC-H7	SK1	BD Biosciences
CD16	PE	3G8	Beckman Coulter
CD56	PE	NCAM16.2	BD Biosciences
HLADR	APC-H7	L243	BD Biosciences
TCRαβ	FITC	WT31	BD Biosciences
TCRγδ	PE	11F2	BD Biosciences
CD45RO	PE-Cy7	UCHL1	BD Biosciences
CD45RA	APC	HI100	BD Biosciences
CD19	PerCP-Cy5.5	SJ25C1	BD Biosciences
CD27	PE	L128	BD Biosciences
IgM	APC	G20-127	BD Biosciences
IgD	FITC	IA6–2	BD Biosciences

To assess antigens, 100 µL of nucleated cell suspension was incubated in each tube in the dark for 20 minutes at room temperature with established mixtures of titered antibodies. One aliquot of cells was incubated with fluorescently labeled monoclonal murine IgG_1_ and IgG_2A_ antibodies (BD Biosciences) directed against nonhuman antigens to determine background fluorescence (isotype antibody negative controls). All samples were subsequently washed with PBS/0.1% NaN_3_ using an automated BD FACS Lyse Wash Assistant (BD Biosciences) prior to acquisition on a flow cytometer. Six-color multiparametric data were acquired and analyzed on a FACSCanto II Flow Cytometer (BD Biosciences) using FacsDiva software (BD Biosciences). Flow rates of 800–1,200 events were used for data acquisition until either 3,000 lymphocyte events, or 3 minutes had elapsed.

Initial flow cytometry gating was performed by isolating all lymphoid cells using forward and side scatter. After initial gating a sequential strategy is used (S3 Fig). Lymphoid cells were additionally gated into CD3+ T cells, CD19+ B cells, or CD3- CD16/56+ NK cells. Further cellular subclassification was performed on separate samples for these individual subsets: CD45RO = CD3/CD45RO, CD45RA = CD3/CD45RA, γδ T cells = CD3/TCR γδ, Memory B cells = CD19+/CD27+, and Class Switched Memory B = CD19+/CD27+/IgM-/IgD-.

For CYTOF, biobanked PBMCs from before and after the first and second doses of the SARS-CoV-2 vaccine were stained with the Fluidigm/Standard Bio Maxpar Direct Immune Profiling assay (containing 30 markers that allows discrimination of 37 cell types). In brief cells were fixed and barcoded for multiplexing, Fc blocked, stained for 30 min at room temperature with the lyophilized pellets of metal conjugated antibodies and fixed again while staining again for 30 minutes with the Fix-Ir Intercalator at room temperature. Mass cytometry was performed with data acquisition on the CyTOF Helios (Fluidigm/Standard BioTools). Population calling was performed using the Maxpar Pathsetter program (Fluidigm/Standard BioTools) which uses probability state modeling rather than manual gating [27].

#### Single-cell RNA-seq.

Thawed PBMCs from pre-vaccination, following 2^nd^ vaccine dose, and following COVID-19 infection underwent CD19 and CD3 enrichment utilizing MicroBeads and LS Columns on the QuadroMACS™ Separator (Miltenyi Biotec). Single-cell suspensions of ~1,200 cells/µl in PBS were prepared for scRNA-seq using 10X Chromium Next GEM Single Cell 5’ Kit v2 also using the Chromium Single Cell Human TCR and BCR Amplification Kits (10x Genomics, Pleasanton, CA). Libraries were constructed per the manufacturers protocol. Libraries were sequenced on an Illumina NovaSeq 6000 with 150 bp paired end reads to ~240 million reads for the gene expression libraries and 80 million reads each for the TCR and BCR libraries (NCBI Gene Expression Omnibus (GEO) accession number GSE266519).

#### Single-cell RNA-seq data processing.

The demultiplexed fastq files were run through the Cell Ranger (v 7.1.0) multi pipeline (10X Genomics) to generate the gene expression count matrix, T cell V(D)J and B-cell V(D)J analysis files. The data files from each sample were then combined and normalized by running through the Cell Ranger aggr pipeline. These aggregated data matrices were then used for downstream analyses. For all portions of the analysis default parameters were used unless noted.

Gene expression data analysis was performed using R (v4.3) and the Seurat package (v5) aligned to hg38 following a standard workflow [28]. In brief, the aggregated filtered feature matrix with 118,950 cells was loaded into a Seurat data object. Poor quality cells with greater than 20% mitochondrial gene expression were filtered out, as well as suspected doublets and empty reactions using nFeature < 200 or nFeature>= 4000. Using the V(D)J metadata, cells with both T- and B-cell clonotypes were considered doublets and removed (966 cells).

After filtering, 117,492 cells remained for downstream analysis. The data were normalized and scaled using SCTransform using default parameters with an additional parameter to regress out percent mitochondrial gene expression. After running PCA with default conditions for dimensionality reduction, cells were visualized using UMAP and clusters were identified using the default FindClusters method and a resolution of 0.4. Cluster identities were predicted using SingleR based on a reference immune cell data set “MonacoImmuneData” [20,29]. Gene markers for each cluster further confirmed the cluster identities. Clusters containing monocytes, dendritic cells, and other non-lymphocytes were removed, after which 105,800 lymphocytes remained.

To avoid the influence of high-expressing B- and T cell receptor clonotypes in visualization and clustering, these genes were removed from the lymphocyte dataset count matrix using the patterns “TR[ABDG][VJC]” or “IG[HJKL]” [30]. The data were renormalized, visualized with UMAP, and clustered as above. Cluster identities were again predicted using SingleR, as above. Then, the data object was subset into separate T cell and B cell analyses.

The two samples taken after COVID positivity were excluded for both T and B cell analyses and only pre- and post-vaccination analyses were conducted. The T cell dataset had 37,988 and 37,469 pre- and post-vaccination cells respectively. The B cell dataset had 13,049 cells total. Cluster identities were predicted using SingleR, as above. For differential gene expression between T cell responders (Good versus Poor), the object was first subset into separate analyses for pre- and post-vaccination. Gene set enrichment was performed using Enrichr [31–34]. TCR and BCR clonotypes were added as metadata to the Seurat objects above using the filtered contig annotations from the VDJ sequencing. Only the primary clonotype was kept per barcode. The immunarch library (v1.0) was used to generate all clonotype summary data as well as identify top clones per patient sample [35]. To identify unique post vaccination clonotypes the top clonotypes were compared for each patient pre and post vaccination. This list was then annotated using the TCRMatch tool to identify epitopes targeted by the TCR clonotypes [21]. Cells mapped to this list of clonotypes were then selected and highlighted in the T cell UMAP divided by T cell response.

### Quantification and statistical analysis

Descriptive statistics were performed. Fisher’s exact tests for categorical variables, Mann-Whitney test for continuous variables unless otherwise noted, and Wilcoxon signed-rank tests were used for paired data were performed, with two-sided p-values ≤ 0.05 considered statistically significant. Gehan-Breslow-Wilcoxon tests were conducted in GraphPad Prism v9.2.0. All statistical analysis details for each experiment can be found in the figure legend of the experiment.

### Highlights

**•** Humoral and adaptive immune responses to SARS-CoV-2 mRNA vaccination were assessed in a cohort of healthy subjects, and autologous and allogeneic transplant patients.
**•** Robust antibody responses were observed in all cohorts post COVID-19 booster vaccination.
**•** The alloSCT cohort had lower rates of SARS-CoV-2 T cell specific response than healthy subjects.
**•** Defects were observed in the central and effector memory T cell populations of alloSCT patients with a poor T cell response to vaccination.

## Supporting information

S1 Fig Additional flow cytometry data. A. Additional clinical pre-vaccination immunodeficiency flow panel results separated based on antibody response of allogeneic patients. B. Additional clinical pre-vaccination immunodeficiency flow panel results separated based on SARS-CoV-2 specific T cell stimulation assay response in allogeneic patients. C. Additional clinical pre-vaccination immunodeficiency flow panel results separated based on cGVHD in allogeneic patients. Mann-Whitney test for continuous variables were performed, with two-sided p-values ≤ 0.05 considered statistically significant.
(PDF)

S2 Fig Additional single cell RNA-seq data. A. Table of characteristics of the individuals profiled by single cell RNA-seq. B. Distribution of Feature Counts per individual broken out by vaccination time point. C. Mitochondrial Read Percentage per individual broken out by vaccination time point. D. Heatmap showing the top three marker genes for each T cell cluster in Fig 4B. E. Cells from the UMAP projection in Fig 4D were split by individual and colored by cluster annotation. F. Gene set enrichment analysis with Enrichr of the differential gene expression between good and poor T cell responders post vaccination (left). Gene Expression of Central Memory T cell markers that are at the top of the differential gene expression table when comparing the gene expression post vaccination of the good versus poor T cell responders (right). These genes are *SELL/CD62L*, *CCR7*, *TCF7*, and *CD27*. G. Data was filtered to include only B cell clusters and excluded two post-COVID-19 infection samples. UMAP projection of the B cells re-clustered and re-annotated as with T cells. H. Heatmap showing the top marker genes for each B cell cluster in S2 Fig 2G I. Unique B cell clonotype number for each individual at each time point.
(PDF)

S3 Fig Immunodeficiency flow cytometry gating strategy. A. Lymphoid Cell gate with subpopulation gating for CD4+ and CD8+ and CD3-CD16/56+. B CD3 gate with subpopulation gating for TCRαβ, TCRγδ, HLADR+, CD45RO and CD45RA. C. CD19 gate with subpopulation gating for CD27+ and CD27+/IgM-/IgD-.
(PDF)

S1 Table Immunosuppression characteristics of allogeneic cohort. (PDF)

S2 Table Multivariate Analysis. Multivariate logistical regression for variables in relation to SARS-CoV-2 Antibody Response.
(PDF)

S3 Table Clinical Characteristics. Clinical characteristics of patients for which CYTOF was performed.
(PDF)

S4 Table Differential gene expression. Differential gene expression results between pre and post vaccination of all cohorts (control, good T cell responders, poor T cell responders), as well between each of the cohorts at both pre and post vaccination timepoints. Includes results of pathway and gene set enrichment analysis from EnrichR for the comparison at the pre-vaccination time point between good and poor responders.
(XLSX)

S5 Table Clonotype Analysis. Tab 1. Top 256 clonotypes unique to post vaccination time point. Tab 2. Results from the TCRMatch database. Tab 3–9. Clonotype per cell for each of the individuals profiled in the single cell analysis.
(XLSX)

S6 Table Summary of patient samples tested per assay. (XLSX)
