# Supplementary figures and images for: Impact of memory T cells on SARS-CoV-2 vaccine response in hematopoietic stem cell transplant
Source: PLoS One. 2025 Apr 28;20(4):e0320744. doi: 10.1371/journal.pone.0320744 (PMC12036906; doi:10.1371/journal.pone.0320744)

**A.**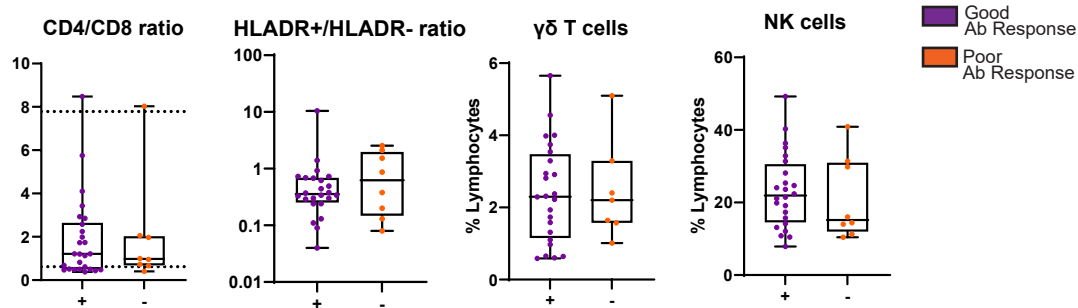**B.**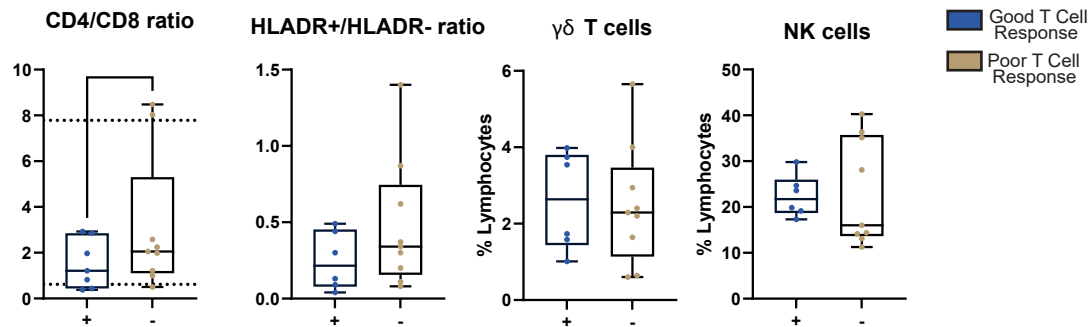**C.**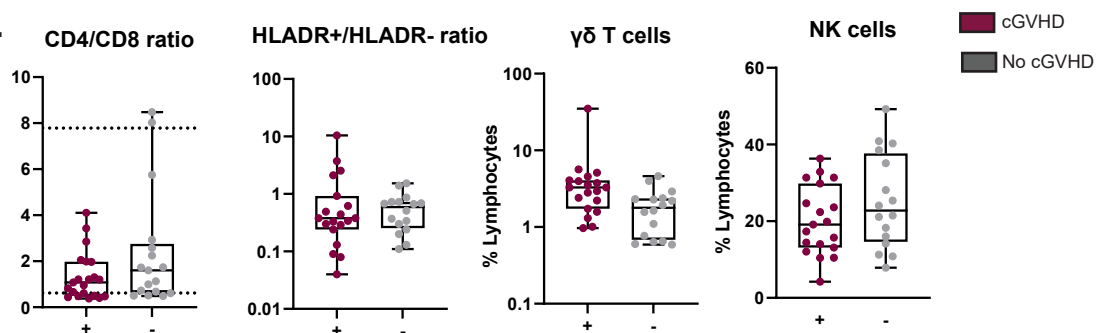

Supplement: S1 Fig — A. Additional clinical pre-vaccination immunodeficiency flow panel results separated based on antibody response of allogeneic patients. B. Additional clinical pre-vaccination immunodeficiency flow panel results separated based on SARS-CoV-2 specific T cell stimulation assay response in allogeneic patients. C. Additional clinical pre-vaccination immunodeficiency flow panel results separated based on cGVHD in allogeneic patients. Mann-Whitney test for continuous variables were performed, with two-sided p-values ≤ 0.05 considered statistically significant. (PDF) [file pone.0320744.s001.pdf]

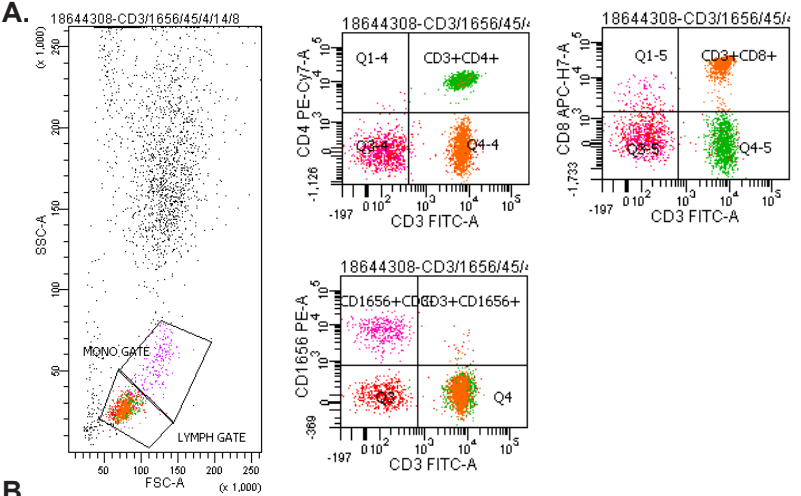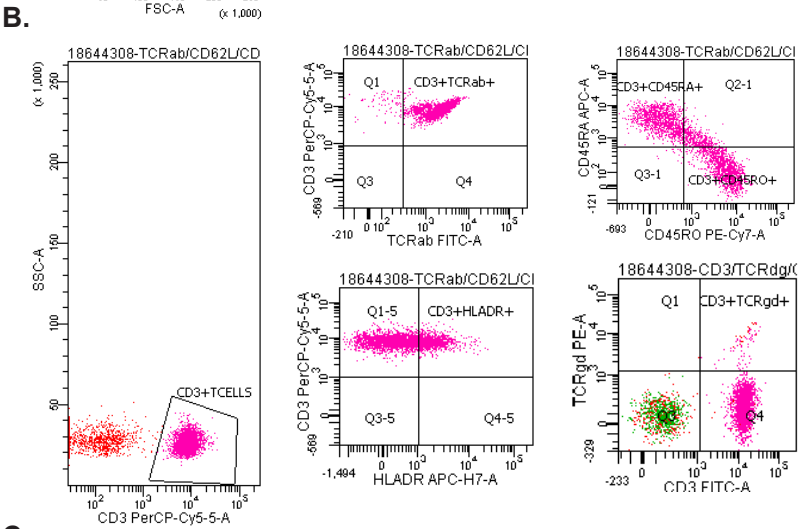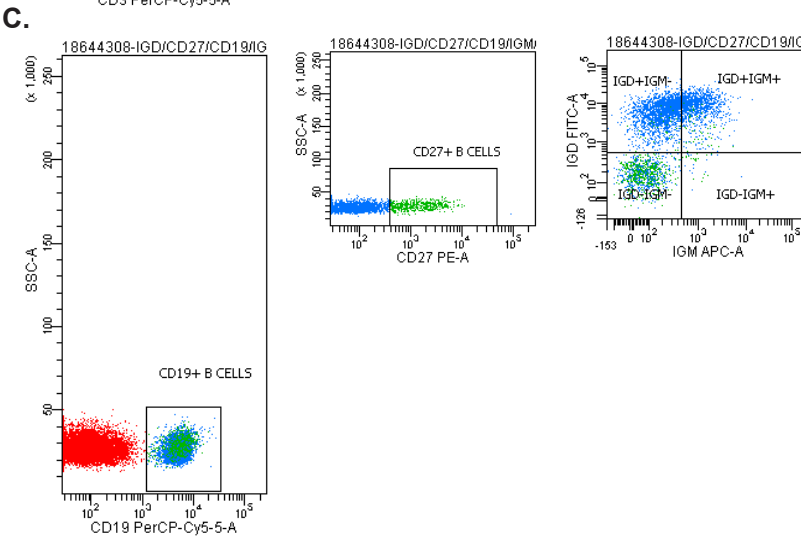

Supplement: S3 Fig — A. Lymphoid Cell gate with subpopulation gating for CD4+ and CD8+ and CD3-CD16/56+. B CD3 gate with subpopulation gating for TCRαβ, TCRγδ, HLADR+, CD45RO and CD45RA. C. CD19 gate with subpopulation gating for CD27+ and CD27+/IgM-/IgD-. (PDF) [file pone.0320744.s003.pdf]
