# Supplementary material for: Impact of memory T cells on SARS-CoV-2 vaccine response in hematopoietic stem cell transplant
Source: PLoS One. 2025 Apr 28;20(4):e0320744. doi: 10.1371/journal.pone.0320744 (PMC12036906; doi:10.1371/journal.pone.0320744)

A.

ID	Gender	Age	Hematologic Malignancy History	TTV (mo)	Allogenic Transplant Type	Post Transplant Cytoxan	aGVHD history	cGVHD Present	Immuno-suppression	GVHD flare after vaccine	GVHD Treatment	Abs CD4+ (count/ul)	Abs CD8+ (count/ul)	Abs CD19+ (count/ul)	Absolute Lymphocyte count (x1000/uL)	Antibody Response/ T Cell Response	Vaccination Brand
P7	F	61	Therapy related AML	12	MRD	No	No	Yes	NA	Yes	Steroid, Tacrolimus	590.4	207	428.4	1.5	Good/Good	Pfizer
P19	F	72	Secondary AML	7	Haplo	Yes	No	No	NA	No	NA	499.8	222.6	278.6	1.4	Good/Poor	Pfizer
P27	F	77	MDS	7	Haplo	Yes	No	No	Steroid	No	NA	45.8	5.4	6	0.2	Poor/Poor	Moderna
P28	F	73	AML	14	MMUD	No	Yes	Yes	Steroid, Tacrolimus	yes	Jakafi	465	568.5	66	1.5	Good/Good	Pfizer
V01	M	36	Healthy Control														Moderna
V02	F	54	Healthy Control														Moderna
V04	M	82	Healthy Control														Moderna

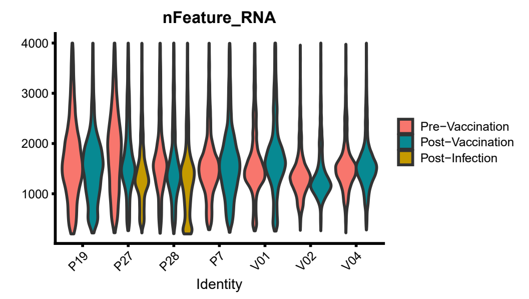

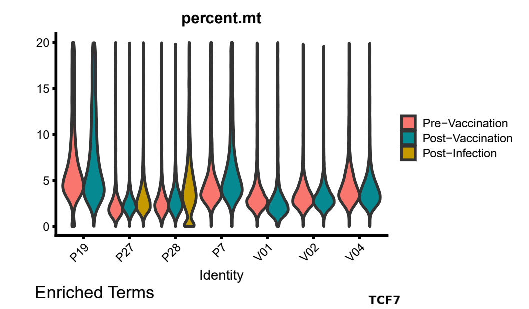

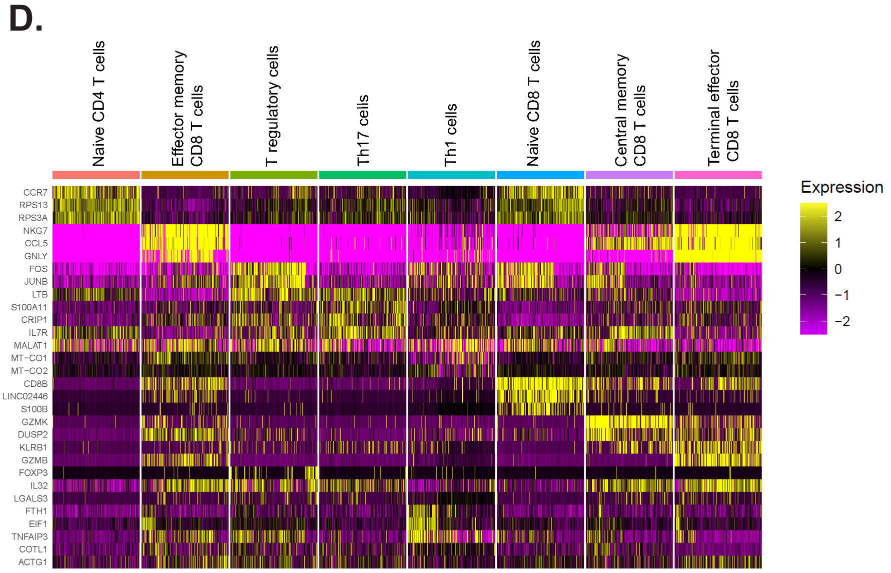

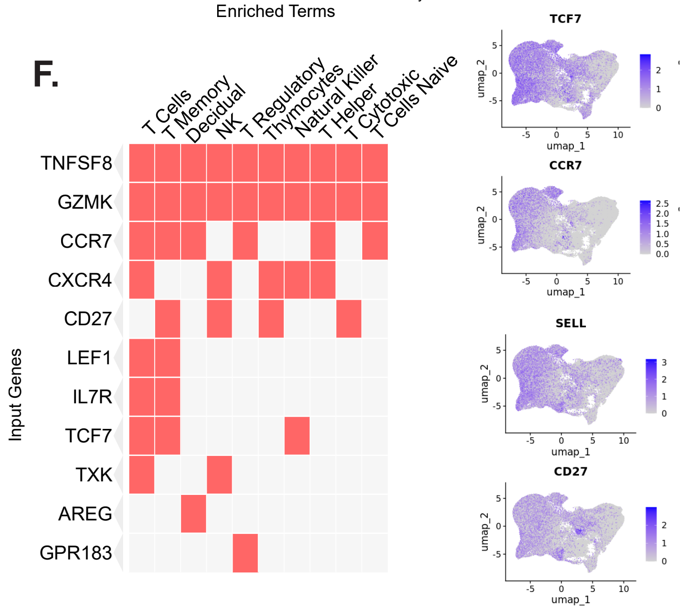

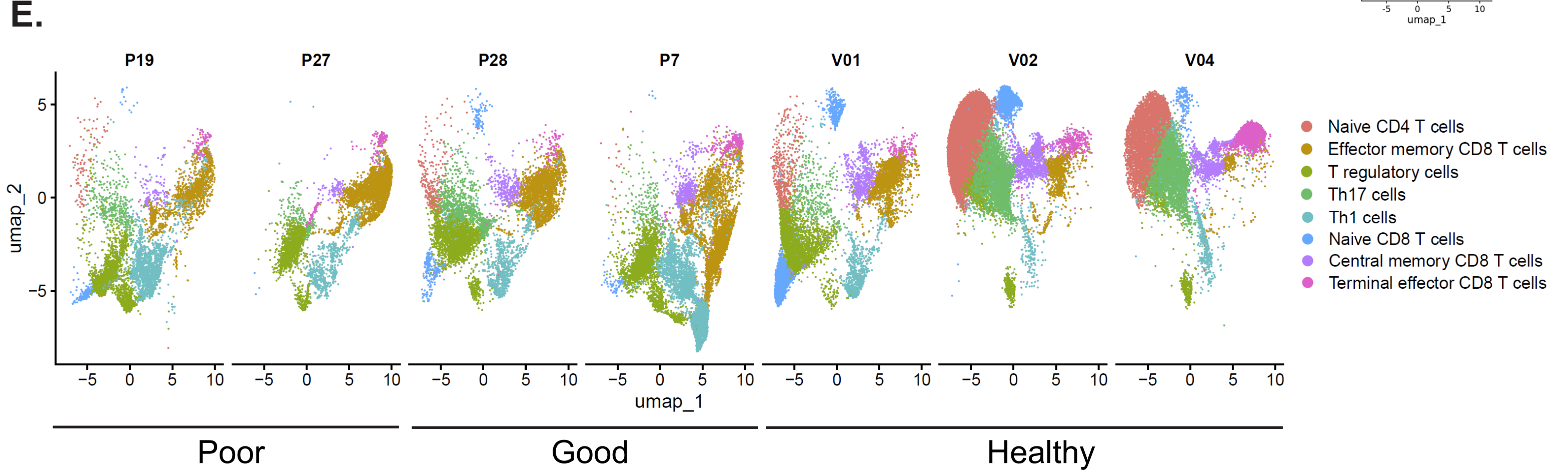

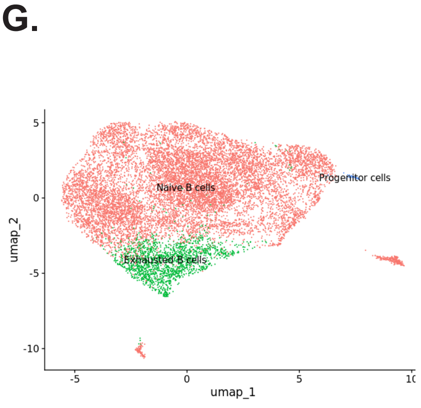

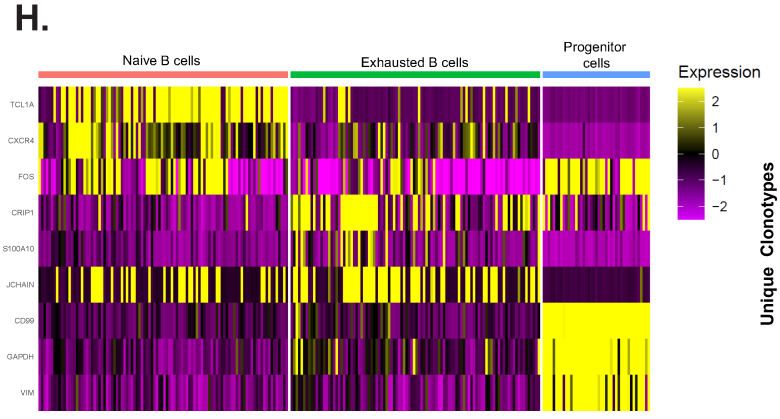

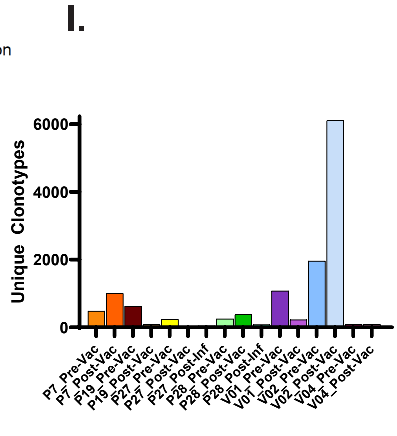

Supplement: S2 Fig — A. Table of characteristics of the individuals profiled by single cell RNA-seq. B. Distribution of Feature Counts per individual broken out by vaccination time point. C. Mitochondrial Read Percentage per individual broken out by vaccination time point. D. Heatmap showing the top three marker genes for each T cell cluster in Fig 4B. E. Cells from the UMAP projection in Fig 4D were split by individual and colored by cluster annotation. F. Gene set enrichment analysis with Enrichr of the differential gene expression between good and poor T cell responders post vaccination (left). Gene Expression of Central Memory T cell markers that are at the top of the differential gene expression table when comparing the gene expression post vaccination of the good versus poor T cell responders (right). These genes are SELL/CD62L, CCR7, TCF7, and CD27. G. Data was filtered to include only B cell clusters and excluded two post-COVID-19 infection samples. UMAP projection of the B cells re-clustered and re-annotated as with T cells. H. Heatmap showing the top marker genes for each B cell cluster in S2 Fig 2G I. Unique B cell clonotype number for each individual at each time point. (PDF) [file pone.0320744.s002.pdf]
