# Supplementary material for: Impact of memory T cells on SARS-CoV-2 vaccine response in hematopoietic stem cell transplant
Source: PLoS One. 2025 Apr 28;20(4):e0320744. doi: 10.1371/journal.pone.0320744 (PMC12036906; doi:10.1371/journal.pone.0320744)
Supplement: S1 Table — (PDF) [file pone.0320744.s004.pdf]

Supplemental Table 1. Immunosuppression of Allogenic Patients

Characteristic	Overall (n=37)	
**Immunosuppression (among allogenic)**		
Any Immunosuppression	20	(54.1)
Steroids	12	(23.4)
Tacrolimus	16	(43.2)
JAK2 inhibitor	3	(8.1)
Photopheresis	3	(8.1)
Multiple immunosuppressants	9	(24.3)
**Therapy (within 3 mo of vaccination)**		
Total	11	(25.6)
Revlimid	3	(7.0)
Daratumumab	1	(2.3)
Carfilzomib	1	(2.3)
Gilteritinib	3	(7.0)
Venetoclax	2	(4.7)
Azacitidine	1	(2.3)
Ibrutinib	1	(2.3)
Receiving IVIG	4	(9.3)
