# Supplementary material for: Impact of memory T cells on SARS-CoV-2 vaccine response in hematopoietic stem cell transplant
Source: PLoS One. 2025 Apr 28;20(4):e0320744. doi: 10.1371/journal.pone.0320744 (PMC12036906; doi:10.1371/journal.pone.0320744)
Supplement: S2 Table — Multivariate logistical regression for variables in relation to SARS-CoV-2 Antibody Response. (PDF) [file pone.0320744.s005.pdf]

Supplemental Table 2. Binary Logistic Regression analysis of variables influencing achievement of good antibody response (Spike IgG > 210 U/mL).

	**p-value**	**Odds Ratio (95% Confidence Interval)**
Age	0.467	0.950 (0.826-1.091)
Post-Transplant Cyclophosphamide	0.428	5.703 (0.077, 422.631)
Time to Vaccination* <12 mo	0.848	0.720 (0.025, 20.628)
History of acute GVHD	0.337	0.244 (0.014, 4.357)
cGVHD at time of vaccine	0.779	2.086 (0.031, 59.897)
Immunosuppression at time of vaccine	0.900	1.363 (0.011, 175.402)
ALC < 1000	0.648	0.383 (0.006, 23.473)
CD4 T cell count < 200	0.950	1.123 (0.031, 41.141)
CD19 B cell count < 50	0.275	1.011 (0.991, 1.032)
CD45RA T cells	0.171	0.916 (0.808, 1.039)
Hypogammaglobulinemia	0.872	1.366 (0.031, 59.897)

*Time to vaccination: time from transplant to first vaccine. ALC: absolute lymphocyte count; CD4
