# Supplementary material for: Impact of memory T cells on SARS-CoV-2 vaccine response in hematopoietic stem cell transplant
Source: PLoS One. 2025 Apr 28;20(4):e0320744. doi: 10.1371/journal.pone.0320744 (PMC12036906; doi:10.1371/journal.pone.0320744)
Supplement: S3 Table — Clinical characteristics of patients for which CYTOF was performed. (PDF) [file pone.0320744.s006.pdf]

Patient #	Age	Diagnosis	Time from transplant (months)	Transplant type	GVHD ppx	active cGVHD at vaccination	Immunosuppression onboard during vaccination
P2	62	AML	20	mismatched unrelated	MTX, Tacrolimus, sirolimus	yes	prednisone, tacrolimus
P4	78	T-PLL	8	matched unrelated	tacrolimus, sirolimus	no	none
P16	64	AML	50	matched related	tacrolimus, sirolimus	no	tacrolimus
P23	65	MDS	16	matched related	tacrolimus, sirolimus	yes	tacrolimus
P25	25	ALL	11	Haplo	tacrolimus, mycophenolate	no	none
P26	66	AML	20	matched related	tacrolimus, sirolimus	yes	tacrolimus
P28	73	AML	14	mismatched unrelated	tacrolimus, sirolimus	yes	prednisone, tacrolimus
P34	75	AML	9	Haplo	MTX, tacrolimus	no	none
